# [^18^F]FE-PE2I DAT correlates with Parkinson’s disease duration, stage, and rigidity/bradykinesia scores: a PET radioligand validation study

**DOI:** 10.1186/s13550-023-00974-7

**Published:** 2023-04-05

**Authors:** Vera S. Kerstens, Patrik Fazio, Mathias Sundgren, Christer Halldin, Per Svenningsson, Andrea Varrone

**Affiliations:** 1 Department of Clinical Neuroscience, Centre for Psychiatry Research, Karolinska Institutet and Stockholm Health Care Services, Stockholm, Sweden; 2 Department of Clinical Neuroscience, Neuro Department, Karolinska Institutet and Karolinska University Hospital, Stockholm, Sweden

**Keywords:** [^18^F]FE-PE2I, DAT, Disease duration, Motor scores, Parkinson’s disease

## Abstract

**Background:**

Correlations between dopamine transporter (DAT) availability and Parkinson’s disease (PD) motor symptoms vary depending on the imaging modality, choice of regions of interest and clinical measures. We aimed to validate the PET radioligand [^18^F]FE-PE2I as a clinical biomarker in PD, hypothesizing negative correlations between DAT availability in specified nigrostriatal regions with symptom duration, disease stage and motor symptom scores.

**Methods:**

We included 41 PD patients (age 45–79 years; H&Y stage < 3) and 37 healthy control subjects in a cross-sectional study with dynamic [^18^F]FE-PE2I PET. Binding potential (BP_ND_) was estimated in the caudate nucleus, putamen, ventral striatum, sensorimotor striatum, and substantia nigra using the cerebellum as reference region.

**Results:**

We found negative correlations (*p* < 0.02) between symptom duration and BP_ND_ in the putamen and sensorimotor striatum (*r*_s_ = − .42; *r*_s_ = − .51), and between H&Y stage and BP_ND_ in caudate nucleus, putamen, sensorimotor striatum, and substantia nigra (*r*_s_ between − .40 and − .54). The first correlations were better described with exponential fitting. MDS-UPDRS-III in ‘OFF’ state correlated negatively (*p* < 0.04) with BP_ND_ in the sensorimotor striatum (*r*_s_ = − .47), and excluding tremor score also in the putamen (*r*_s_ = − .45).

**Conclusion:**

Results are in agreement with earlier findings in in vivo and post-mortem studies and validate [^18^F]FE-PE2I as a functional PD biomarker for PD severity.

*Trial registration*: EudraCT 2011-0020050, Registered April 26 2011; EudraCT 2017-003327-29, Registered October 08 2017; EudraCT 2017-001585-19, Registered August 2 2017. https://eudract.ema.europa.eu/.

**Supplementary Information:**

The online version contains supplementary material available at 10.1186/s13550-023-00974-7.

## Background

Idiopathic Parkinson’s disease (PD) is a slowly progressing neurodegenerative disorder affecting the nigrostriatal dopaminergic system [1]. Diagnosis is often delayed, and no disease-modifying treatment is available. Sensitive diagnostic biomarkers are therefore needed to facilitate earlier diagnosis and assess potential disease-modifying treatment, as well as enable patient stratification and monitor disease progression.

Molecular imaging studies of the dopaminergic system have contributed to the evidence that the measured degree of neurodegeneration of nigrostriatal dopaminergic projections [2, 3, 4, 5, 6, 7, 8, 9, 10, 11] is related to the severity of motor symptoms. However, dopamine transporter (DAT) imaging studies do not suggest a strong correlation between DAT availability and clinical motor symptoms [12]. Since the majority of the DAT studies have been performed with single-photon emission computer tomography (SPECT), this might in part be related to the suboptimal resolution of the imaging technique that does not permit detailed mapping of the DAT in striatal subregions. The striatum is anatomically divided into putamen (involved in the control of movements) and caudate﻿ nucleus (more involved in cognition). The striatum can also be divided *functionally* into motor, associative, and limbic striatal subregions [13, 14]. The higher resolution of positron emission tomography (PET) provides a more sensitive way to measure such smaller regions or lower density regions and thus provides a better tool to measure neurodegeneration patterns in Parkinson’s disease and assess its relationship to clinical symptom scores.

The reason to choose PET radioligand [^18^F]FE-PE2I is that it has high DAT affinity and selectivity, beneficial kinetics over the close analogue [^11^C]PE2I, and good reliability and repeatability [15, 16, 17, 18, 19].

This study aimed to validate [^18^F]FE-PE2I PET measures as a clinical biomarker in PD, by assessing its correlations with symptom duration, disease stage, and severity of motor symptoms in PD patients with non-advanced disease. Non-advanced PD was chosen, based on the evidence from the literature that in later disease stages DAT decline levels reach a plateau. Correlation analyses with MDS-UPDRS-III scores (Movement Disorder Society—Unified Parkinson’s Disease Rating Scale motor scores) were performed including and excluding tremor scores, based on the knowledge that bradykinesia and rigidity are associated more strongly than tremor with DAT availability in motor-specific striatal subregions [20].

Based on previous findings, our hypothesized regions of interest (ROIs) for negative correlation with symptom duration were sensorimotor striatum and substantia nigra; for correlation with Hoehn & Yahr (H&Y) stage were caudate﻿ nucleus, putamen, and sensorimotor striatum; and for correlation with MDS-UPDRS-III score were putamen and sensorimotor striatum. For exploratory purposes, correlations were performed also with the remaining striatal regions, as well as with the regions in the less and more affected hemisphere specifically.

## Methods

Consecutive cohorts were combined from three different studies of the same principal investigator in which the same inclusion and exclusion criteria, imaging protocols, and PET system were used, and a total of three investigators for the clinical scales were involved. Based on power calculations using previous findings on DAT, a sample of 40 patients should be able to measure a correlation coefficient of 0.4 with power 0.73 (*α* = 0.05). The studies were approved by the Swedish Ethical Review Authority, the Radiation Safety Committee of the Karolinska University Hospital, and the Swedish Medicinal Product Agency (EudraCT 2011-0020050, EudraCT 2017-003327-29, and 2017-001585-19). Written informed consent was given by the participants. The study was conducted in accordance with the ethical standards of the Declaration of Helsinki.

Demographic, clinical, and baseline PET data of the first cohort of PD patients (*n* = 20) have been previously reported [21]. The second cohort (*n* = 27) includes a group of eleven PD patients that were examined in a previously reported test–retest study [15] and sixteen PD patients that were prospectively recruited.

### Study population and clinical data collection

Patients with idiopathic PD, age 45–80, and H&Y stage < 3, were recruited at two local outpatient clinics for movement disorders and through advertisement at the Swedish Parkinson Foundation. Cognitive impairment was excluded through Mini-Mental State Exam (MMSE) ≥ 27; psychiatric comorbidity through structured psychiatric interview; clinically significant physical comorbidity through physical examination, ECG, routine urine and blood tests; significant intracranial deviations through structural MRI. Additional file 2: Table S1 shows summarized exclusion criteria and rules of conduct minimizing possible confounding factors during DAT measurements.

Symptom duration was approximated from the reported onset of motor symptoms. The modified Hoehn and Yahr staging scale was used. In the first cohort, Unified Parkinson’s Disease Rating Scale motor score (UPDRS-III) had been performed at the screening visit while on Parkinson medication for practical reasons. In the remaining patients, the Movement Disorder Society—Unified Parkinson’s Disease Rating Scale motor score (MDS-UPDRS-III) was used and assessed while in practically defined ‘OFF’ state on the PET day, i.e. at least 12 h off dopaminergic medication, or 24 h in case of the longer-lasting Parkinsonian medication. This last improvement in the protocol was implemented to be able to perform correlation analyses between clinical measures and DAT availability in ‘OFF’ state, considering the effect of Parkinsonian medication on the motor scores.

Healthy controls (HC) were recruited and sex and age-matched (± 5 years) to the included patients with Parkinson’s disease, to correct on a group level for age- and sex-related effects on DAT availability when comparing disease-related differences to healthy controls.

### MRI acquisition

T1- and T2-weighted images were acquired with a 3 Tesla MRI system (General Electric, Discovery MR750). MRI acquisition was done as previously described [15, 21], see Additional file 1 for details. The T1-weighted images were used for co-registration with PET images and delineation of the regions of interest (ROI); the T2-weighed images were used to exclude clinically significant pathology.

### PET acquisition and reconstruction

All PD subjects included were in practically defined ‘OFF’ state during PET acquisition. An individually made plaster helmet was used for head fixation in the PET camera. [^18^F]FE-PE2I was produced in-house as previously described [22]. PET acquisition and reconstruction were done as previously described [15]. In short, list mode PET data were acquired for 93 min with a high-resolution research tomograph (HRRT, Siemens Molecular Imaging). A 6-min transmission scan using a ^137^Cs source was performed before radioligand administration for attenuation correction. PET data were reconstructed into 37 frames of increasing duration (8 × 10, 5 × 20, 4 × 30, 4 × 60, 4 × 180, 12 × 360 ms) using 3D OP OSEM with 10 iterations and 16 subsets, including modelling of the point spread function, with a transaxial resolution of ~ 2 mm [23]. The first two minutes of data were used as reference image for frame-to-frame realignment. Frame-to-frame realignment was performed using statistical parametric mapping (SPM, version 12). Realignment plots were evaluated visually to exclude excessive head motion. In the case of translations of more than 3 mm in the z-axis, additional motion correction was performed.

### Data analysis

**Fig. 1.**
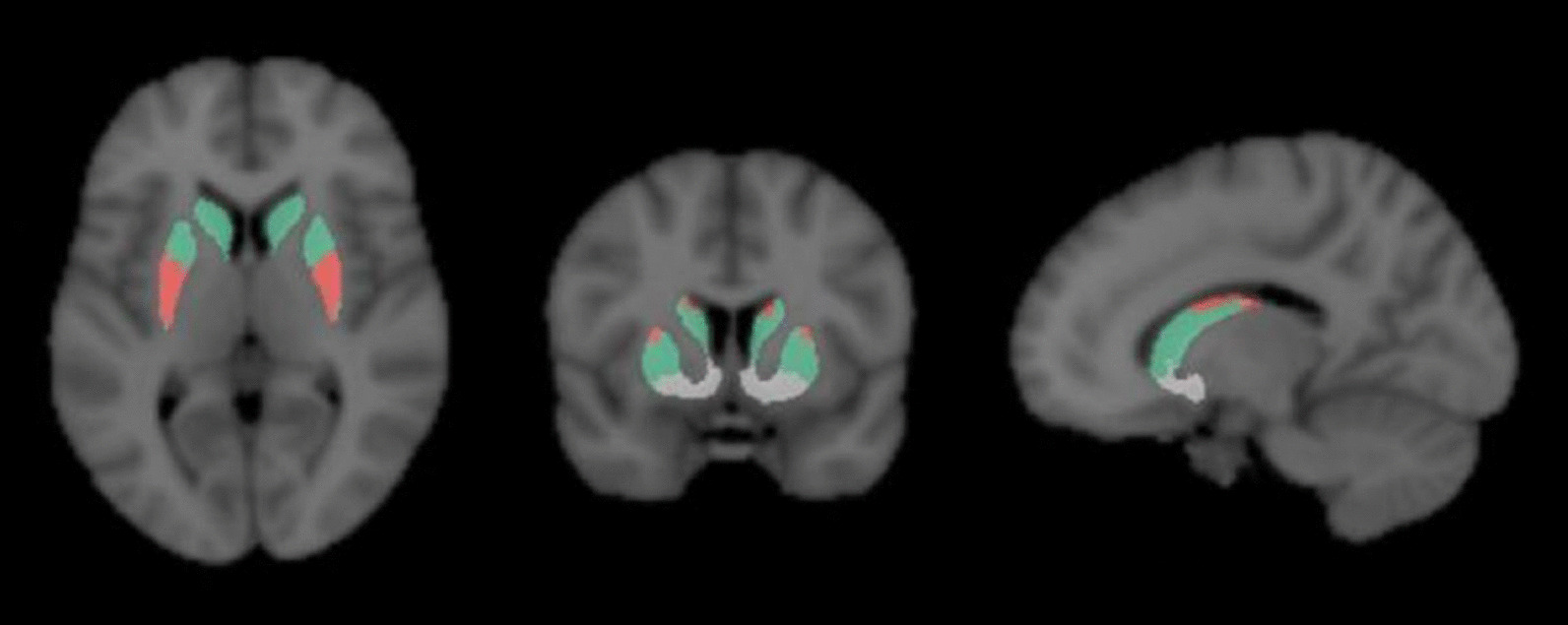
3D Functional subdivisions of striatum using the FSL-template overlaid on T1-weighted MRI sequences, with the sensorimotor striatum displayed in red. Figure previously published as Fig. 1 in Kerstens et al., Clin Transl Imaging 2020. Republication permitted under CC BY licensing

The analysis plan was pre-registered at https://aspredicted.org/f3rq6.pdf. Three-dimensional (3D) regions of interest were automatically delineated on the T1-weighted MR image and co-registered to PET. The main striatal regions striatum, caudate﻿ nucleus, putamen, and accumbens area (in this manuscript referred to as *ventral striatum; VS*) were delineated using Freesurfer. An FSL template (https://fsl.fmrib.ox.ac.uk/fsl/fslwiki/Atlases/striatumconn) was used for the delineation of the sensorimotor striatum (Fig. 1), and an in-house developed template [21] was used for the delineation of the substantia nigra.

DAT availability (BP_ND_) was estimated with wavelet-aided parametric imaging (t* = 27 min), using the Logan graphical analysis and cerebellum as reference region, as described earlier [15]. BP_ND_ (binding potential) is calculated as distribution volume ratio (DVR) minus 1: DVR-1.

ROI sides were assigned as belonging to the more or less affected hemisphere based on the individual’s BP_ND_ in the putamen sides, with the lower putamen BP_ND_ being assigned as the more affected hemisphere (maff), and the higher BP_ND_ as the less affected hemisphere (laff). These sides were cross-checked with the laterality of the MDS-UPDRS-III scores.

### Statistics

Statistical analysis was performed with R version 3.6.2. The normality of distribution was tested with the Shapiro–Wilk test and density plots. Testing for the difference in variance between the PD and control group was done with Levene’s test. Identification of statistically significant outliers was tested with Grubb’s test, combined with boxplots for visual assessment. The difference between the mean BP_ND_ of control subjects and PD subjects was tested with an unpaired, one-sided *t* test. The calculation of the effect size of BP_ND_ differences between controls and PD patients was done with Cohen's ds, with the formula: (mean(controls)-mean(PD))/SD(controls and PD).

The asymmetry index (AI) in PD was calculated per ROI as:

$$\left( {{\text{BP}}_{{{\text{ND}}}} {\text{laff}} - {\text{BP}}_{{{\text{ND}}}} {\text{maff}}} \right)/\left( {{\text{average}}\;{\text{BP}}_{{{\text{ND}}}} {\text{laff}}\;{\text{ and}}\;{\text{ maff}}} \right)*{1}00.$$

As reference asymmetry index, the upper limit of the 95% CI of the control group was used. The 95% CI was calculated as follows:

$${\text{mean}}\left( {{\text{absolute}}\;{\text{AI}}\;{\text{ROI}}\left[ {{\text{Controls}}} \right]} \right)\, \pm \,qt\left( {t,{\text{df}}\, = \,\left( {n - {1}} \right)} \right)*\left( {\left( {{\text{sd}}\left( {{\text{absolute }}\;{\text{AI}}\;{\text{ ROI}}\left[ {{\text{Controls}}} \right]} \right)/{\text{sqrt}}\left( n \right)} \right)} \right),$$

$${\text{with}}\;t\, = \,0.{975}\;(t\, = \,{\text{alpha}}\, + \,\left( {{1} - {\text{alpha}}} \right)/{2}).\, {\text{Alpha}}\, = \,0.{95}\;{\text{and}}\;n\, = \,{37}.$$

which takes the sample distribution into account instead of assuming a normal distribution.

Simple linear regression was used for the correlation analysis between DAT availability and clinical measures, as described in the pre-registration. In the case of categorical or ordinal variables (such as H&Y stage), a generalized linear model (GLM) was used. LEDD (levodopa equivalent daily dose), age, and sex were tested as covariates.

A priori hypotheses had a *p* value threshold of < 0.05 for statistical significance; other comparisons were adjusted for multiple comparisons with Meff-correction [24, 25].

### Data sharing

Data are available upon reasonable request.

## Results

Forty-seven PD subjects and thirty-nine age- and sex-matched control subjects were included in the study. Data from six patients and two controls were excluded because of the following reasons: Subject Without Evidence of Dopaminergic Deficit (SWEDD) *n* = 2; problematic positioning in camera *n* = 4; PET stopped early upon subject’s request *n* = 2). This left a sample of 41 PD subjects and 37 controls included in the final analysis. Demographics and clinical data of these subjects are presented in Table 1.

**Table 1:** Demographic and clinical data of the study population

	Sex	Age (year) (median, range)	Symptom duration (year) (median, range)	LEDD (mg) (median, range)	(MDS-) UPDRS-III (mean ± SD) [*minus tremor score]*	H&Y stage (median; range)
PD Total, *n* = 41	12F/29M (29/71%)	67 (45–79)	3 (0.25–14)	400 (0–1010)	–	–
Subgroup 1* (’ON’)	*5F*/*13M (28/72%)*	*65 (46–71)*	*2.5 (0.25–12)*	*300 (0–940)*	*20.44* ± *6.85 [NA]*	*1.5 (1–2.5)*
Subgroup 2** (‘OFF’)	*7F/16M (30/70%)*	*68 (45–79)*	*3 (1–14)*	*450 (0–1010)*	*21.7* ± *10.7 [17.7* ± *10.1]*	*1 (1–3)*
HC Total, * n* = 37	11F/26M (30/70%)	65 (43–74)	–	–	–	–

The italics are meaningful to show a difference with the first line which is the total summary data, and the two italic rows are subtotals of that one

*Two were excluded compared to the published cohort (Fazio et al., 2018, *n* = 20): one SWEDD and one due to Freesurfer image processing error

**In this subgroup, MDS-UPDRS-III was assessed in practically defined ‘OFF’

### Differences in BP_ND_ between PD and control subjects

**Fig. 2:**
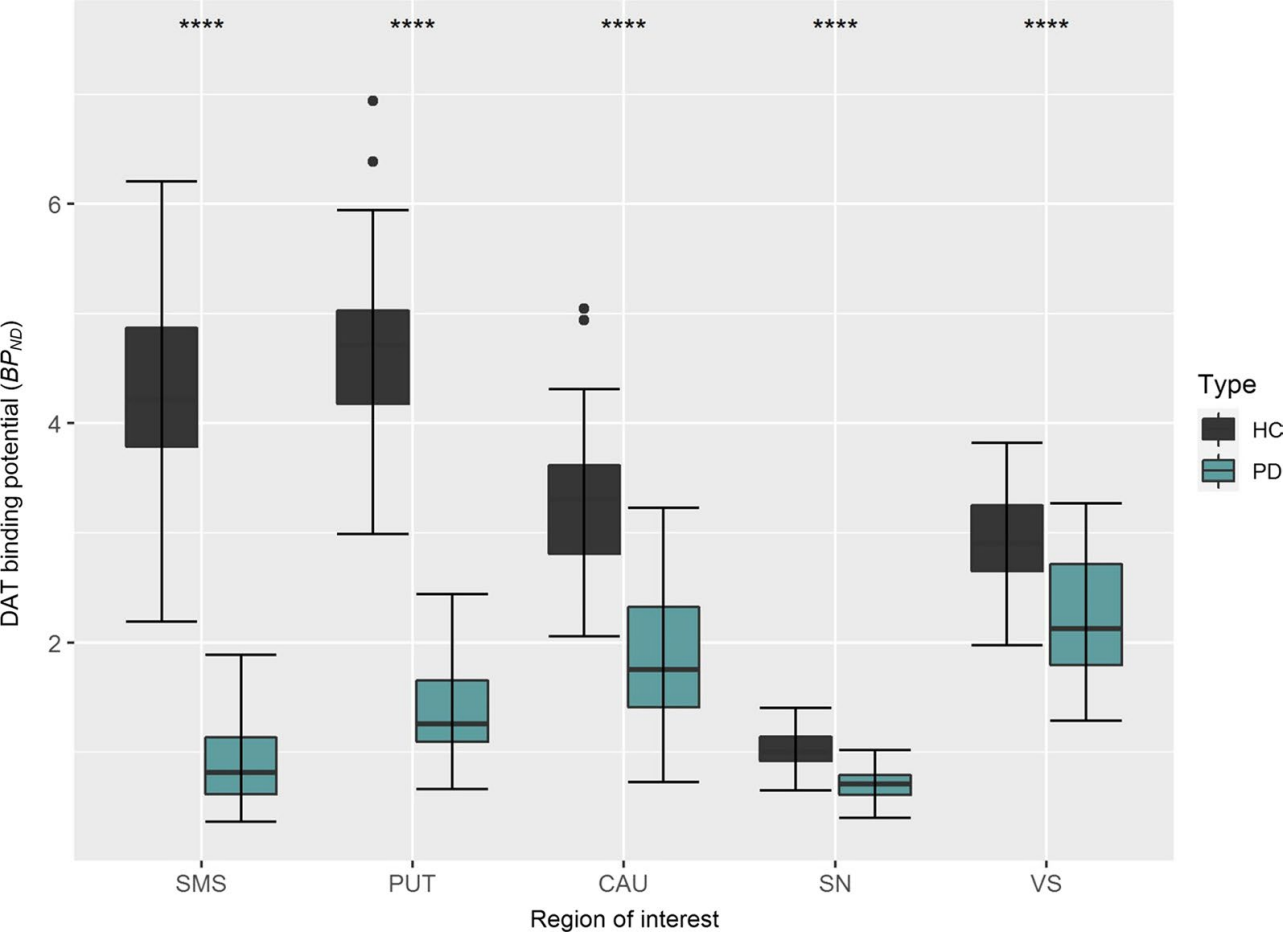
Binding potential (BP_ND_) values of [^18^F]FE-PE2I in different regions of the nigrostriatal dopaminergic system in HC and PD subjects, presented in order from biggest to smallest inter-group difference. ****p value < .0.0001. STR: striatum; CAU: caudate﻿ nucleus; PUT: putamen; VS: ventral striatum; SMS: sensorimotor striatum; SN: substantia nigra

In PD patients, the mean total striatal DAT availability (BP_ND_*)* was 60% lower than in controls (see Fig. 2; Table 2). The largest difference was observed in the sensorimotor striatum and putamen (79% and 71% lower than controls), followed by the caudate﻿ nucleus (45%), and the substantia nigra and ventral striatum (31% and 25% lower than controls). Effect sizes for the distinction of PD from controls ranged between 1.18 (ventral striatum) and 1.86 (putamen) (Table 2). The putamen-to-caudate ratio was 0.79 ± 0.20 in PD patients and 1.44 ± 0.16 in controls. The ventral striatum-to-caudate ratio was 1.32 ± 0.40 in PD patients and 0.91 ± 0.17 in controls.

**Table 2:** Regional [^18^F]FE-PE2I *BP*_*ND*_ values and corresponding effect sizes in PD versus HC

Brain region	PD *n* = 41 (mean ± SD)	HC *n* = 37 (mean ± SD)	*p* value	Effect size	Asymmetry index (%)	
					PD (median; range)	HC (95% CI)
Striatum	1.61 ± 0.49	4.01 ± 0.69	< 2.2e−16	1.79	24.7; 5.4*–*64.9	2.5*–*4.4
Caudate﻿ nucleus	1.81 ± 0.64	3.31 ± 0.68	8.7e−16	1.50	18.8; 0.7*–*87.1	4.5*–*7.9
Putamen*	1.36 ± 0.44	4.68 ± 0.80	< 2.2e−16	1.86	30.9; 3.6*–*74.8	1.9*–*3.5
Ventral striatum (VS)	2.21 ± 0.54	2.93 ± 0.45	5.6e−09	1.18	7.1; -20.5*–*49.1	9.8*–*14.2
Sensorimotor striatum (SMS)*	0.87 ± 0.34	4.24 ± 0.89	< 2.2e−16	1.85	32.4; -17.6*–*90.0	9.2*–*13.6
Substantia nigra (SN)	0.69 ± 0.14	1.01 ± 0.17	2.0e−14	1.45	16.6; -28.0*–*43.7	7.2*–*11.4

*Non-equal variance between PD and HC groups. *P* value was calculated with a one-tailed, independent *t* test. *PD* subjects with Parkinson’s disease, *HC* healthy controls, *CI* confidence interval

In PD patients, we observed that the lower putamen *BP*_ND_ side indeed corresponded to the contralateral most affected MDS-UPDRS-III side in all except one case. This case had similar *BP*_*ND*_ values in right and left putaminal ROIs, as well as identical left/right total scores of the MDS-UPDRS-III. For this reason, the asymmetry indexes (AI) were calculated excluding this patient (Table 2, Additional file 2: Table S3). The regions with the largest mean AI were the sensorimotor striatum and the putamen (around 35–40%). The mean AI in the caudate﻿ nucleus and substantia nigra was less than 20% and in the ventral striatum less than 10%. However, we observed a wide range of AI values for some of the regions, particularly in the ventral striatum, sensorimotor striatum, and substantia nigra, including negative AI values. Additional analysis on the asymmetry index was, therefore, performed excluding the cases with a negative asymmetry index or in which the asymmetry index was below the upper limit of the reference asymmetry index (see Table 2). With these criteria, the striatum and putamen were the regions in which all PD subjects (*n* = 40) had an asymmetry index above the threshold, whereas in the sensorimotor striatum, substantia nigra, and ventral striatum, this was the case for only 88%, 58, and 28% of the PD sample, respectively (Additional file 2: Tables S2, S3).

### Correlations between DAT and motor symptoms

**Fig. 3:**
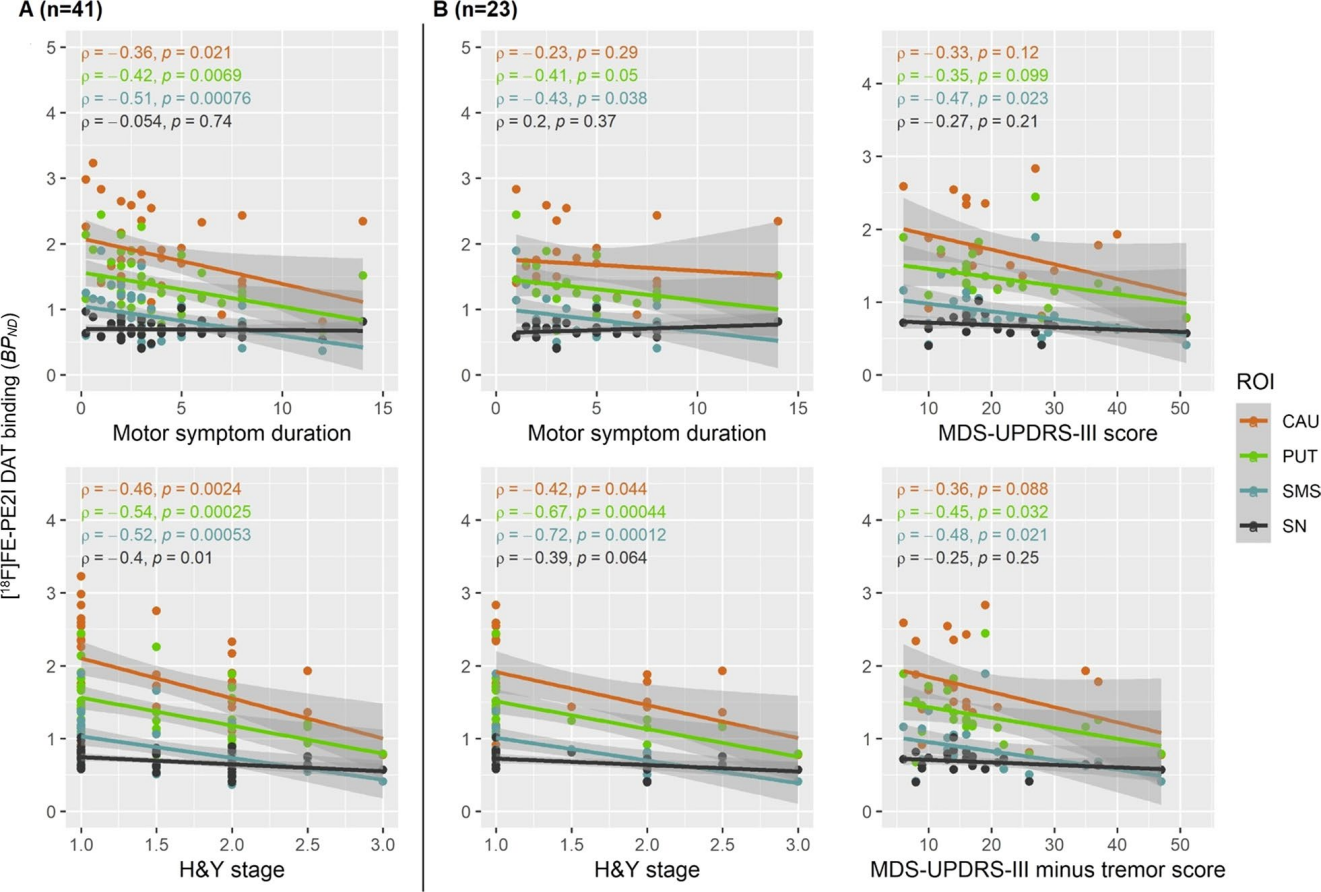
Correlations of the three clinical measures with the hypothesized and exploratory nigrostriatal regions combined, in the total group (*n* = 41, column **A**) and the clinically assessed ‘OFF’ subgroup (*n* = 23 two columns of **B**). *ρ*: Spearman’s rho; ROI: region of interest; CAU: caudate﻿ nucleus; PUT; putamen; SMS; sensorimotor striatum; SN: substantia nigra. *P* value cut-off for post hoc analysed regions 0.019 (correcting for multiple comparisons for four regions with calculated meff: 0.05/2.63)

The relationships between symptom duration, H&Y stage, and MDS-UPDRS-III scores and [^18^F]FE-PE2I BP_ND_ in the a priori-defined regions and additional exploratory regions are presented in Fig. 3. Correlational analysis with MDS-UPDRS-III was only done with the PD subgroup that had this measure assessed in practically defined ‘OFF’, i.e. *n* = 23. Regarding relationships in a priori-defined regions, statistically significant negative correlations were found for symptom duration and DAT availability in the sensorimotor striatum, but not in the substantia nigra. H&Y stage and DAT correlated in all hypothesized regions (caudate﻿ nucleus, putamen, substantia nigra), and even in substantia nigra; in the subgroup of patients assessed in ‘OFF’, only a trend was observed between BP_ND_ in substantia nigra and H&Y stage. MDS-UPDRS-III score correlated negatively only with BP_ND_ in the sensorimotor striatum, but when subtracting the tremor from the total score, the correlation was significant also in the putamen.

**Fig. 4:**
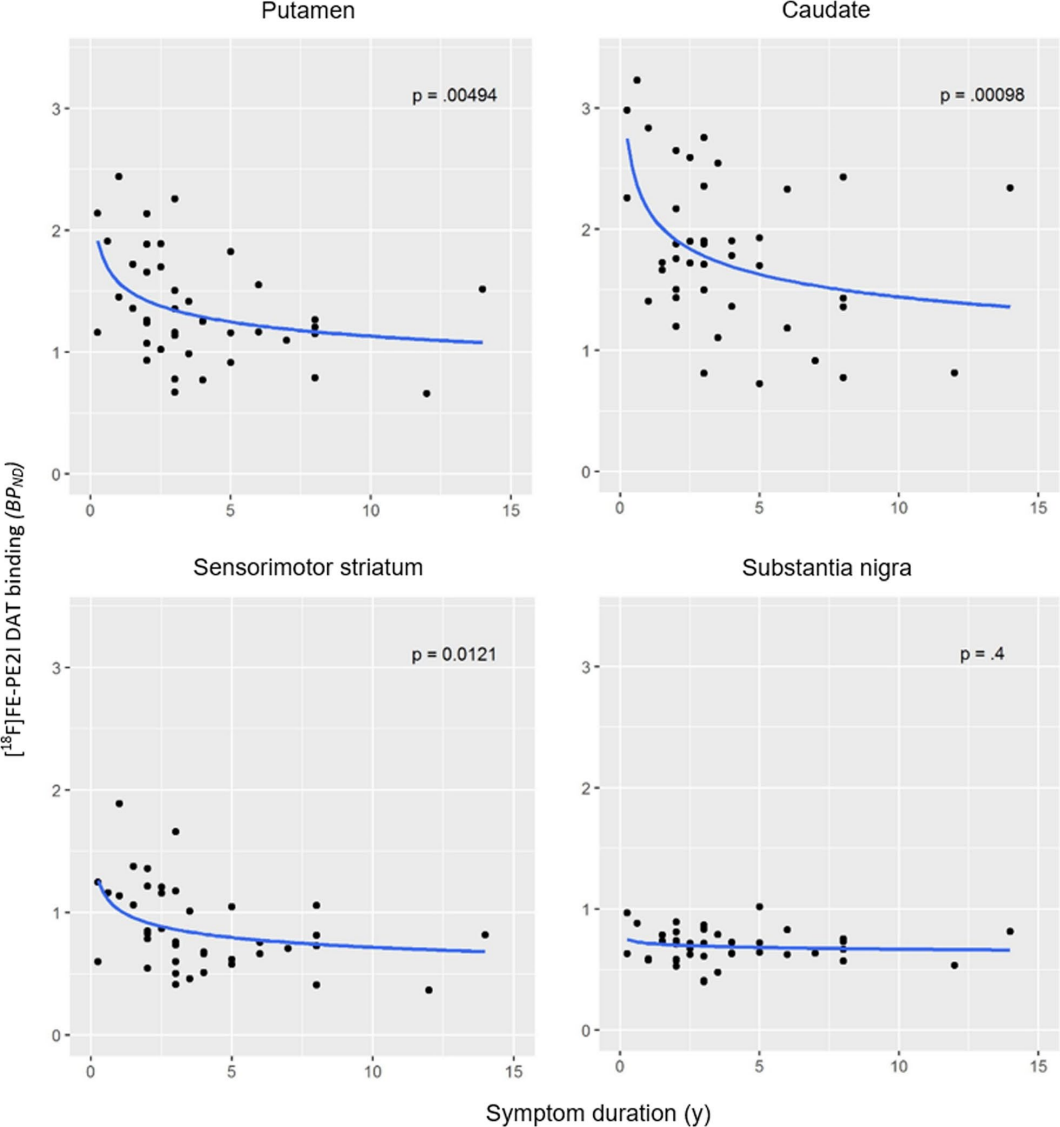
Exponential fits of the relationship between symptom duration and [^18^F]FE-PE2I BP_ND_ in putamen, caudate﻿ nucleus, sensorimotor striatum, and substantia nigra (*n* = 41)

The relationship between symptom duration and BP_ND_ in caudate﻿ nucleus and putamen was better described using an exponential function (Fig. 4). Exponential fitting was not superior to linear fitting for the relationship between MDS-UPDRS-III and the DAT availability in the four main ROIs (results not shown).

In post hoc analyses, significant negative correlations were also found for symptom duration and DAT availability in putamen (Fig. 3, *n* = 41), less affected putamen and less affected sensorimotor striatum (Additional file 2: Table S3a/b). Correlations between the H&Y stage and DAT availability in less affected caudate﻿ nucleus, more and less affected putamen, and more and less affected sensorimotor striatum were also statistically significant in the post hoc analysis (Additional file 2: Table S3a). No additional statistically significant negative correlations of MDS-UPDRS-III scores with any of the other areas were found.

## Discussion

This study was designed to validate [^18^F]FE-PE2I PET as a biomarker for dopaminergic neurodegeneration in non-advanced PD, through a cross-sectional study on its correlations with clinical symptom severity scores. To increase the statistical power compared with an earlier study [21], we enlarged the sample size by including an additional cohort of PD patients with similar clinical characteristics. Patients in this cohort were evaluated with MDS-UPDRS-III in practically defined ‘OFF’ state on the day of the PET measurement so that correlations between motor scores and DAT availability were performed while patients were examined under the same conditions (OFF). Furthermore, correlations were performed using the MDS-UPDRS-III score including and excluding the tremor score, with the knowledge that bradykinesia and rigidity are symptoms more strongly associated with striatal DAT availability than tremor. Additionally, correlations with clinical measures were evaluated in functional subdivisions of the striatum.

### Pattern in dopaminergic neurodegeneration

[^18^F]FE-PE2I BP_ND_ data in the whole group of 41 PD patients were compared to those obtained in a group of 37 control subjects with similar age and sex distribution. To our knowledge, this is the largest reported cohort of PD patients examined with [^18^F]FE-PE2I PET. We confirmed our earlier findings of a PD-related pattern of DAT deficit and extended the quantification also to the sensorimotor striatum. Overall, the findings of this study corroborate the knowledge that dopaminergic neurodegeneration in PD follows a retrograde pattern, affecting neuronal terminals first (striatum) before the cell bodies (substantia nigra) [21]. Within the striatum, the posterior putamen (*sensorimotor striatum*) is affected first, followed by the anterior putamen, caudate﻿ nucleus, and ventral striatum.

### BP_ND_ in the substantia nigra

In the substantia nigra, there was no clear asymmetry of BP_ND_ between the less and more affected hemispheres. DAT density in the substantia nigra is much lower than the DAT in the striatal regions, and we have also previously shown that in the substantia nigra BP_ND_ estimates have lower reliability than in the striatal regions, making accurate measurement of DAT in this area challenging. Neuronal loss in the substantia nigra is mainly observed in the pars compacta, with the ventrolateral tier being relatively spared [26]. The substantia nigra ROI template that we used in this study covers the entire region and is based on the distribution of uptake of [^18^F]FE-PE2I [22]. Therefore, it is possible that we were not able to detect an asymmetry of BP_ND_ because DAT was measured in the whole region rather than in the part of the substantia nigra that is most severely affected. Partial volume effect on the small area might have influenced the results as well. In a previous study with [^11^C]PE2I PET in mild-to-moderate PD patients [20], BP_ND_ was measured in subdivisions of the substantia nigra. Although it was found that the DAT deficit was larger in the ventral tier than the dorsal tier (*p* < 0.001) [20], no significant difference in BP_ND_ was observed between the more vs. less affected substantia nigra, in agreement with the findings of this study.

### Correlations with clinical measures

In the publication of the first cohort [21], no statistically significant correlation was found between BP_ND_ and disease duration, H&Y, or UPDRS motor score. Our improved sample size and clinical data collection protocol enabled a more powered assessment of the correlations.

In agreement with other previous DAT imaging studies [12, 27], we observed a significant negative correlation between symptom duration and DAT availability measured with [^18^F]FE-PE2I in the putamen, and more specifically the sensorimotor striatum. This correlation was not significantly influenced by the inclusion of the patient with a statistical outlier symptom duration (14 years). The relation between symptom duration and DAT availability in the putamen, caudate﻿ nucleus, and sensorimotor striatum was better described by exponential fitting than linear fitting. This non-linear relationship between symptom duration and dopaminergic deficit has been previously described in a large cohort of PD patients examined with the vesicular monoamine transporter type 2 PET radioligand [^18^F]FP-DTBZ [28]. The findings are not surprising, since it is known that clinical signs of Parkinson’s disease do not appear until about 50–70% of the nerve terminals are already lost [26, 29, 30, 31, 32]. This might result in larger inter-individual variability of the dopaminergic deficit at the onset of the disease, with a more uniform decline as the disease progresses. The exponential correlation suggests that early on in the disease course, there is some degree of variability of DAT availability that probably reflects the fact that patients develop symptoms at different levels of dopaminergic deficit, probably due to different motor or dopamine reserve.

A significant negative correlation was found between the H&Y stage and [^18^F]FE-PE2I BP_ND_ in all nigrostriatal regions (caudate﻿ nucleus, putamen, and substantia nigra). The H&Y stage is a measure of the patient’s global motor disability. These findings suggest that the H&Y stage is the clinical measure that is most directly related to the overall nigrostriatal dysfunction, compared to the other clinical variables used.

A significant negative correlation between the MDS-UPDRS-III score and DAT availability in the putamen was only found when the tremor score was subtracted from the total score. It has to be highlighted, though, that one subject presented BP_ND_ values that were relatively high compared to their MDS-UPDRS-III score (Fig. 3), was identified as an outlier by the Grubb’s test, and indeed influenced the statistical results significantly. Methodological reasons for this subject’s relatively high BP_ND_ values were not found, and, therefore, the subject was not excluded from the analysis, despite being an outlier from a statistical point of view. Exploratory re-analysis with the removal of this subject gave correlations of BP_ND_ with total MDS-UPDRS-III scores in the putamen and sensorimotor striatum both with linear fitting (r_s_ between -0.43 and -0.56, p values between 0.006 and 0.05, see Additional file 2: Fig. S1a), as well as with exponential fitting (p-values < 0.05, see Additional file 2: Fig. S1b).

Previous [^18^F]FE-PE2I studies in PD patients have reported no significant correlations between striatal DAT and UPDRS-III [21, 33]. Possible explanations for these differences might be related to whether UPDRS-III was measured ‘ON’ or ‘OFF’ medication, or whether the motor score included also the tremor score or only the bradykinesia and rigidity scores. Two PET studies performed with the close analogue [^11^C]PE2I found that striatal DAT-binding significantly correlated with total UPDRS-III (OFF) and bradykinesia-rigidity scores [3] or with bradykinesia, rigidity, and axial symptoms, but not with tremor score [20]. Overall, the findings of this study support the latter and suggest that the tremor score should be subtracted from the total MDS-UPDRS-III score in studies that intend to examine the correlation between DAT and motor symptoms.

There was no correlation of DAT availability in the substantia nigra with any of the clinical measures in patients examined ‘OFF’ medication, thereby not confirming our initial hypothesis. A possible explanation is that in the early stages of PD, there is still a relative sparing of neurodegeneration in substantia nigra, and the relationship might be more clearly expected later on in the disease process. Furthermore, a floor effect due to the low density of DAT could also be an explanation for the lack of correlation findings. However, in the combined group (*n* = 41), the correlation between BP_ND_ in the substantia nigra and H&Y stage became statistically significant likely due to increased sample size.

### Correlation with the clinical measure of less vs. more affected hemisphere

When examining the correlation between BP_ND_ in unilateral regions and symptom duration and H&Y stage, statistically significant correlations were found in those regions that also displayed significant correlation in the bilateral BP_ND_ (main analysis). In the case of MDS-UPDRS-III scores, no statistically significant correlations were found with more or less affected ROI sides specifically.

## Conclusions

This PET study with [^18^F]FE-PE2I enabled measurement of the degree of DAT deficit in patients with non-advanced PD in different regions of the nigrostriatal system. Furthermore, it enabled examination of the correlations between clinical measures and DAT availability in these regions. The results show that symptom duration correlates non-linearly with striatal DAT availability, supporting the notion that DAT deficit is highly variable when motor symptoms start developing and approaches a plateau when the disease has progressed. The H&Y stage was found to correlate with DAT availability in all nigrostriatal regions, suggesting that this measure more closely reflects the overall dopaminergic dysfunction than the other clinical measures used. The correlation with MDS-UPDRS-III scores was significant in both putamen and sensorimotor striatum when subtracting the tremor score, confirming that DAT availability in the motor striatum is more strongly associated with bradykinesia and rigidity than tremor symptoms. Overall, the findings validate the PET radioligand [^18^F]FE-PE2I as an in vivo marker for PD severity.

## Supplementary Information

**Additional file 1.** Supplementary material.

**Additional file 2.** Supplementary tables and figures.

## Data Availability

The dataset of the current study is available from the corresponding author on reasonable request.

## Abbreviations

DAT: Dopamine transporter
PD: Parkinson’s disease
PET: Positron emission tomography
^[18F]^FE-PE2I: 18F-(E)-*N*-(3-iodoprop-2-enyl)-2b-carbofluoroethoxy-3b-(49-methyl-phenyl) nortropane
H&Y (stage): Hoehn and Yahr stage
BP_ND_: Binding potential, DAT availability
(MDS-)UPDRS-III: (Movement Disorder Society—)Unified Parkinson’s Disease Rating Scale motor scale
SPECT: Single-photon emission computer tomography
ROI: Region of interest
MMSE: Mini-Mental State Exam
ECG: Electrocardiogram
MRI: Magnetic Resonance Imaging
HC: Healthy controls
3D OP OSEM: Three-dimensional ordered Poisson ordered subset expectation–maximization
SPM: Statistical parametric mapping
FSL: FMRIB's Software Library
Maff: More affected hemisphere
Laff: Less affected hemisphere
SD: Standard deviation
AI: Asymmetry index
CI: Confidence interval
GLM: Generalized linear model
LEDD: Levodopa equivalent daily dose
Meff: Estimate the effective number of tests
SWEDD: Subject without evidence of dopaminergic deficit

## References

[1] Kalia LV, Lang AE (2015). Parkinson’s disease. Lancet (London, England).

[2] Nandhagopal R (2008). Progression of dopaminergic dysfunction in a LRRK2 kindred: A multitracer PET study. Neurology.

[3] Li W (2018). 11C-PE2I and 18F-Dopa PET for assessing progression rate in Parkinson’s: a longitudinal study. Mov Disord.

[4] Benamer HTS (2000). Correlation of Parkinson’s disease severity and duration with 123I-FP-CIT SPECT striatal uptake. Mov Disord.

[5] Eshuis SA, Maguire RP, Leenders KL, Jonkman S, Jager PL (2006). Comparison of FP-CIT SPECT with F-DOPA PET in patients with de novo and advanced Parkinson’s disease. Eur J Nucl Med Mol Imaging.

[6] Ishikawa T, et al. Comparative nigrostriatal dopaminergic imaging with iodine-123-βCIT-FP/SPECT and fluorine-18-FDOPA/PET. J Nucl Med. 1996 Nov;37(11):1760–5. 8917170

[7] Shih MC, et al. Neuroimaging of the dopamine transporter in Parkinson’s disease: first study using [99mTc]-TRODAT-1 and SPECT in Brazil. Arq Neuropsiquiatr. 2006;64(3A):628–34. 10.1590/s0004-282x2006000400021 17119808

[8] Ottaviani S (2006). Comparative analysis of visual and semi-quantitative assessment of striatal [123I]FP-CIT-SPET binding in Parkinson’s disease. Neurol Sci.

[9] Pirker W. Correlation of dopamine transporter imaging with parkinsonian motor handicap: How close is it? Mov Disord. 2003;18 Suppl 7:S43–51. 10.1002/mds.10579 14531046

[10] Seibyl JP (1995). Decreased single-photon emission computed tomographic {123I}β-CIT striatal uptake correlates with symptom severity in parkinson’s disease. Ann Neurol.

[11] Varrone A, Marek KL, Jennings D, Innis RB, Seibyl JP (2001). 123I]β-CIT SPECT imaging demonstrates reduced density of striatal dopamine transporters in Parkinson’s disease and multiple system atrophy. Mov Disord.

[12] Kaasinen V, Vahlberg T (2017). Striatal dopamine in Parkinson disease: A meta-analysis of imaging studies. Ann Neurol.

[13] Cervenka S, Bäckman L, Cselényi Z, Halldin C, Farde L (2008). Associations between dopamine D2-receptor binding and cognitive performance indicate functional compartmentalization of the human striatum. Neuroimage.

[14] Tziortzi AC (2011). Imaging dopamine receptors in humans with [11C]-(+)-PHNO: dissection of D3 signal and anatomy. Neuroimage.

[15] Kerstens VS, et al. Reliability of dopamine transporter PET measurements with [18F]FE-PE2I in patients with Parkinson’s disease. EJNMMI Res. 2020;10(1):95. 10.1186/s13550-020-00676-4 PMC7427674 32797307

[16] Sasaki T (2012). Quantification of dopamine transporter in human brain using PET with 18F-FE-PE2I. J Nucl Med.

[17] Suzuki M, et al. Reproducibility of PET measurement for presynaptic dopaminergic functions using L-[β-11C]DOPA and [18F]FE-PE2I in humans. Nucl Med Commun Wolters Kluwer Heal Nucl Med Commun. 2014;35(3):231-7. 10.1097/MNM.0000000000000052. 10.1097/MNM.0000000000000052 24468851

[18] Varrone A, et al. In vitro autoradiography and in vivo evaluation in cynomolgus monkey of [18F]FE-PE2I, a new dopamine transporter PET radioligand. Synapse. 2009;63(10):871–80. 10.1002/syn.20670 19562698

[19] Varrone A (2011). Kinetic analysis and quantification of the dopamine transporter in the nonhuman primate brain with 11C-PE2I and 18F-FE-PE2I. J Nucl Med.

[20] Martín-Bastida A (2019). Relationship between neuromelanin and dopamine terminals within the Parkinson’s nigrostriatal system. Brain.

[21] Fazio P, et al. Nigrostriatal dopamine transporter availability in early Parkinson’s disease. Mov Disord. 2018;33(4):592–9. 10.1002/mds.27316 29436751

[22] Stepanov V (2012). An efficient one-step radiosynthesis of [18F]FE-PE2I, a PET radioligand for imaging of dopamine transporters. J Label Compd Radiopharm.

[23] Varrone A, Sjöholm N, Eriksson L, Gulyás B, Halldin C, Farde L. Advancement in PET quantification using 3D-OP-OSEM point spread function reconstruction with the HRRT. Eur J Nucl Med Mol Imaging. 2009;36(10):1639–50. 10.1007/s00259-009-1156-3 19437012

[24] Derringer, J. A simple correction for non-independent tests. 2018. 10.31234/OSF.IO/F2TYW.

[25] Li J, Ji L (2005). Adjusting multiple testing in multilocus analyses using the eigenvalues of a correlation matrix. Heredity.

[26] Fearnley JM, Lees AJ (1991). Ageing and parkinson’s disease: Substantia nigra regional selectivity. Brain.

[27] Strafella AP (2017). Molecular imaging to track Parkinson’s disease and atypical parkinsonisms: new imaging frontiers. Mov Disord.

[28] Hsiao IT (2014). Correlation of Parkinson disease severity and 18F-DTBZ positron emission tomography. JAMA Neurol.

[29] Greffard S (2006). Motor score of the unified Parkinson disease rating scale as a good predictor of lewy body-associated neuronal loss in the substantia nigra. Arch Neurol.

[30] Guttman M (1997). 11C]RTI-32 PET studies of the dopamine transporter in early dopa- naive Parkinson’s disease: Implications for the symptomatic threshold. Neurology.

[31] Ma SY, Röyttä M, Rinne JO, Collan Y, Rinne UK (1997). Correlation between neuromorphometry in the substantia nigra and clinical features in Parkinson’s disease using disector counts. J Neurol Sci.

[32] Tabbal SD (2012). Low nigrostriatal reserve for motor Parkinsonism in nonhuman primates. Exp Neurol.

[33] Jakobson Mo, S. et al. Dopamine transporter imaging with [18F]FE-PE2I PET and [123I]FP-CIT SPECT—a clinical comparison. EJNMMI Res. 2018;8(1):100. 10.1186/s13550-018-0450-0 PMC6238014 30443684

