# Supplementary material for: [18F]FE-PE2I DAT correlates with Parkinson’s disease duration, stage, and rigidity/bradykinesia scores: a PET radioligand validation study
Source: EJNMMI Res. 2023 Apr 5;13:29. doi: 10.1186/s13550-023-00974-7 (PMC10076455; doi:10.1186/s13550-023-00974-7)
Supplement: Supplementary file 1 — Additional file 1. Supplementary material. [file 13550_2023_974_MOESM1_ESM.docx]

# Supplementary material

MRI acquisition parameters, as described in Kerstens et al 2020:

3D T1-weighted sequence has 176 slices of 1mm thickness, field of view 256 × 256 mm, resolution 1 × 1 × 1 mm, inversion time 450 ms, echo time 3.18 ms, and repetition time 8.16 ms.
