# Supplementary material for: [18F]FE-PE2I DAT correlates with Parkinson’s disease duration, stage, and rigidity/bradykinesia scores: a PET radioligand validation study
Source: EJNMMI Res. 2023 Apr 5;13:29. doi: 10.1186/s13550-023-00974-7 (PMC10076455; doi:10.1186/s13550-023-00974-7)
Supplement: Supplementary file 2 — Additional file 2. Supplementary tables and figures. [file 13550_2023_974_MOESM2_ESM.docx]

Supplementary tables and figures

**Table S1**

| **Exclusion criteria (summarized)** | **Rules of conduct before** **PET measurements** |
| --- | --- |
| Any other relevant neurologic or psychiatric disease.  Use of psychotropic medication (e.g. SSRI)  History of severe allergic reaction  Claustrophobia or postural deviations making the MRI or PET procedure impossible  Cardiovascular comorbidities (e.g. (symptomatic) orthostatic hypotension)  Comorbidity judged clinically significant as judged by investigator (e.g. obesity, cancer) | Withdrawal of dopaminergic medication >12h; >24h for dopamine agonists and MAO-B inhibitors  No high-intense exercise during 4 days before PET  No alcohol 48h before PET  No caffeine in 3 hours before PET  No nicotine on the day of PET |

**Table S2**. The asymmetry index (%) of [^18^F]FE-PE2I *BP*_ND_ in PD patients was calculated excluding those cases in which the value was negative or below the threshold established in control subjects.

|  | | STR | CAU | PUT | VS | SMS | SN |
| --- | --- | --- | --- | --- | --- | --- | --- |
| Using AI above the upper limit of 95% CI of the HC | mean ± SD  range | 26.1(14.7)  5.3—65.2 | 25.8% ±15.4  9.4—84.1 | 34.9% ±19.8  3.6—74.3 | 23.6% ±9.8  14.5—49.5 | 44.7% ±24.9  14.8—94.7 | 28.5% ±8.8  14.4—43.8 |
|  | *n=* | *40* | *31* | *40* | *11* | *35* | *23* |
| **REFERENCES** | | | | | | | |
| Fazio et al., MovDis 2018, n=20  Mean ± SD | | - | 16.4% ±29 | 33.2% ±29 | 18.0% ±29 | - | 15.8% ±23 |

| **Table S3a. Correlations between [^18^F]FE-PE2I DAT binding in more and less effected sides of nigrostriatal regions and symptom duration in PD cohort with asymmetry.** | | | | | | | | | |
| --- | --- | --- | --- | --- | --- | --- | --- | --- | --- |
|  | **Caudate** | | **Putamen** | | **Sensorimotor striatum** | | **Substantia nigra** | | |
|  | **maff** | **laff** | **maff** | **laff** | **maff** | **laff** | **maff** | | **laff** |
| **N=40 (total)** | | | | | | | | | |
| **Symptom duration** | r_s_ -.35 | r_s_ -.38 | r_s_ -.3 | **r_s_ -.44*** | r_s_ -.28 | **r_s_ -.53*** | r_s_ -.01 | r_s_ -.03 | |
| **N=22 (‘OFF’)** | | | | | | | | | |
| **Symptom duration** | r_s_ -.14 | r_s_ -.25 | r_s_ -.23 | r_s_ -.42 | r_s_ -.04 | r_s_ -.46 | r_s_ +.14 | r_s_ +.24 | |
| **HY** | r_s_ -.39 | **r_s_ -.54*** | **r_s_ -0.58*** | r_s_ **-0.63*** | r_s_ **-.61 *** | r_s_ **-.72*** | r_s_ -.28 | r_s_ -.4 | |
| **MDS-UPDRS-III OFF**  incl. tremor | r -.31 | r -.39 | r -.22 | r -.36 | r_s_ -.34 | r_s_ -.45 | r -.08 | r -.41 | |
| excl. tremor | r_s_ -.31 | r_s_ -.37 | r_s_ -.25 | r_s_ -.42 | r_s_ -.19 | r_s_ -.46 | r_s_ -.06 | r_s_ -.34 | |

|  | *positive r* |  | *-0-0.2* |  | *-0.2-0.4* |  | *-0.4-0.7* |  | *-0.7-0.9* |  | *-0.9-1.0* |
| --- | --- | --- | --- | --- | --- | --- | --- | --- | --- | --- | --- |

r value color

| **Table S3b. Correlations between [^18^F]FE-PE2I DAT binding in more and less effected sides of nigrostriatal regions and symptom duration in PD cohort with asymmetry, applying HC 95% CI cut-off.** | | | | | | | | | |
| --- | --- | --- | --- | --- | --- | --- | --- | --- | --- |
|  | **Caudate** | | | **Putamen** | | **Sensorimotor striatum** | | **Substantia nigra** | |
|  | **maff** | **laff** | | **maff** | **laff** | **maff** | **laff** | **maff** | **laff** |
|  | *n=31/40* | | | *n=40/40* | | *n=35/40* | | *n=23/40* | |
| **Symptom duration** | *r_s_ -.45* | ***r_s_ -.49**** | | *See Table S3a* | | *r_s_ -.31* | ***r_s_ -.54**** | *r_s_ +.25* | *r_s_ +.13* |
|  | *n=17/22* | | | *n=22/22* | | *n=19/22* | | *n=11/22* | |
| **Symptom duration** | r_s_ -.13 | r_s_ -.29 | *See Table S3a* | | | r_s_ 0.0 | r_s_ -.45 | r_s_ +.48 | r_s_ +.55 |
| **HY** | r_s_ -.44 | r_s_ -.57 | *See Table S3a* | | | r_s_ -.57 | **r_s_** **-.72*** | r_s_ +.22 | r_s_ -.21 |
| **MDS-UPDRS-III OFF**  incl. tremor | r -.33 | r -.41 | *See Table S3a* | | | r_s_-.28 | r_s_ -0.2 | r +.23 | r -.05 |
| excl. tremor | r_s_ -.26 | r_s_ -.35 | *See Table S3a* | | | r_s_ -.12 | r_s_ -.27 | r_s_ +.19 | r_s_ -.06 |

*Table S3a/b notes: r_s_=Spearman rho, r=Pearson r; aff. = affected; laff= less affected side. In bold with a star (*) the statistical significant correlations (p<.010). P-value threshold corrected for multiple comparisons with calculated meff for the 8 regions (4.97): .05/4.97=.010.*

| **Figure S1a. Linear correlation plots of [^18^F]FE-PE2I binding in hypothesized regions with MDS-UPDRS-III (OFF)** **incl. and excl. tremor score when outlier subject removed (n=22)** | | |
| --- | --- | --- |
| [^18^F]FE-PE2I DAT binding *(BP_ND_)* | Putamen  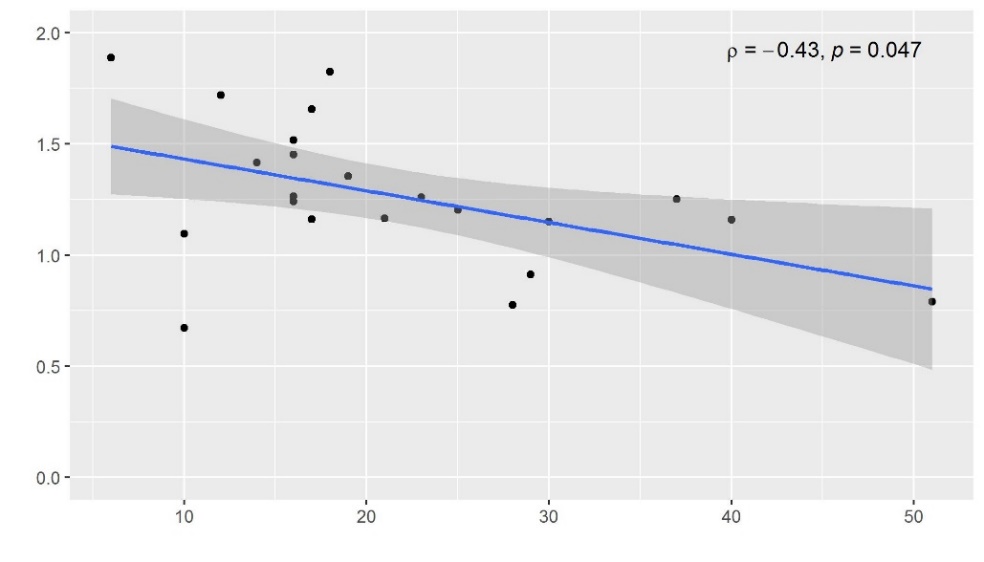 | Putamen  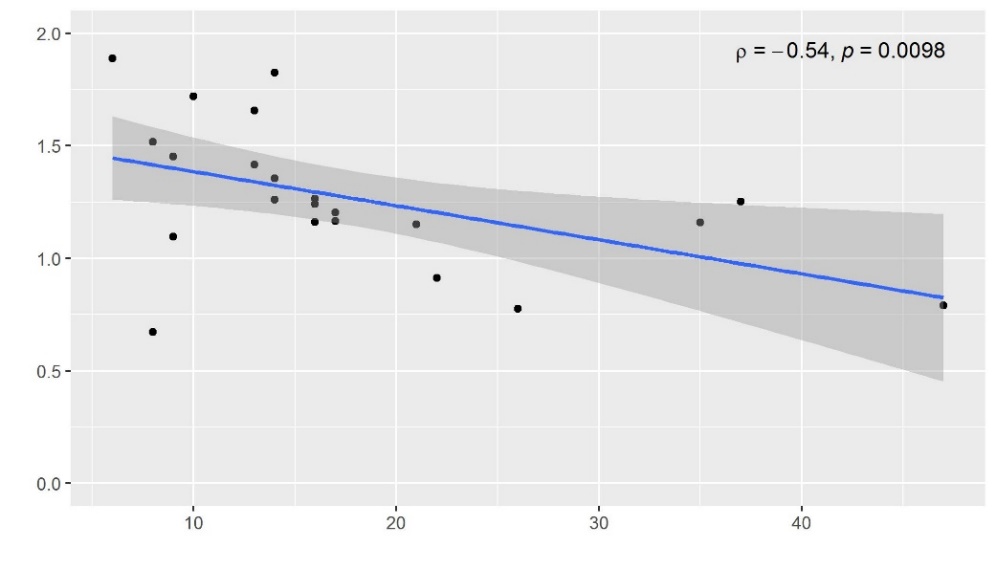 |
|  | SMS  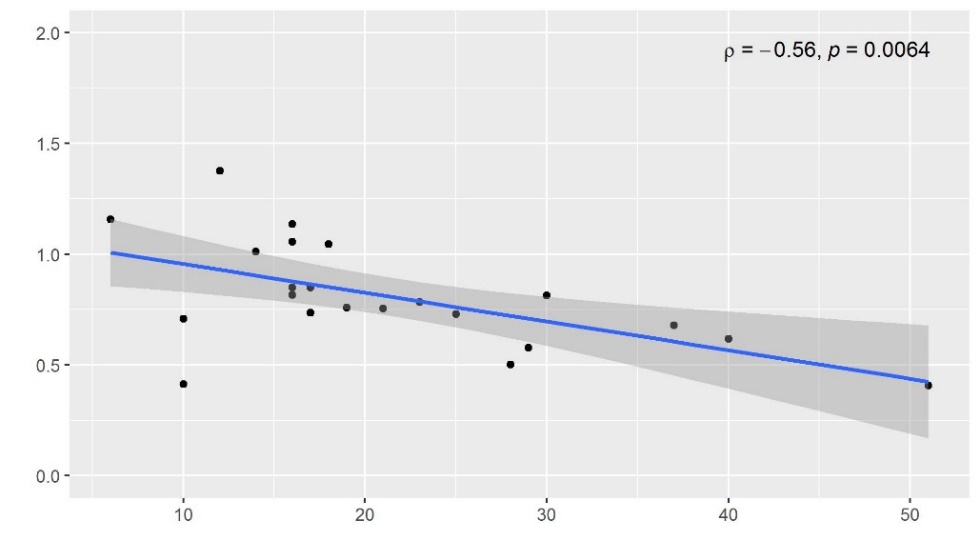 | SMS  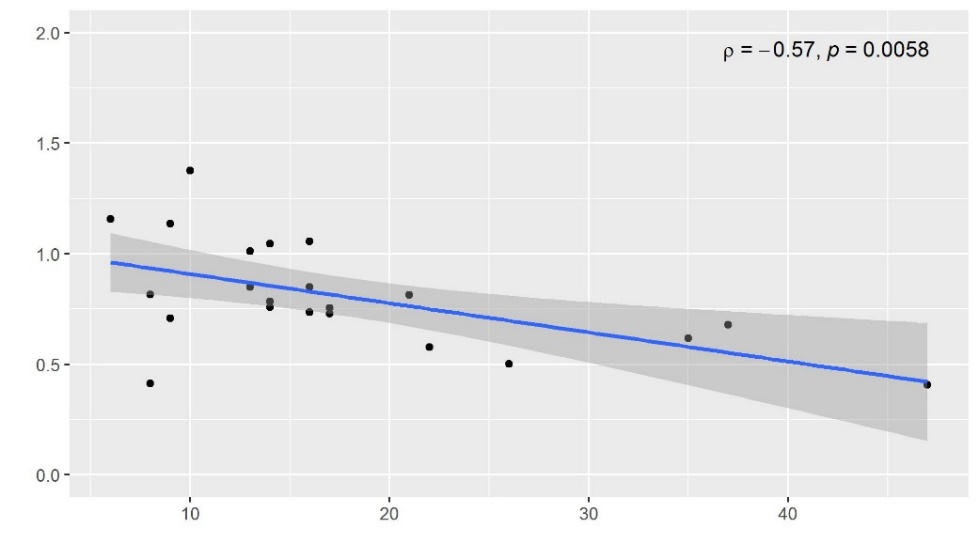 |
|  | **MDS-UPDRS-III score incl. tremor** | **MDS-UPDRS-III score excl. tremor** |

| **Figure S1b. Exponential correlations of [^18^F]FE-PE2I binding with MDS-UPDRS-III incl. and excl. tremor score when outlier subject removed (n=22)** | | | |
| --- | --- | --- | --- |
| [^18^F]FE-PE2I DAT binding *(BP_ND_)* | Putamen  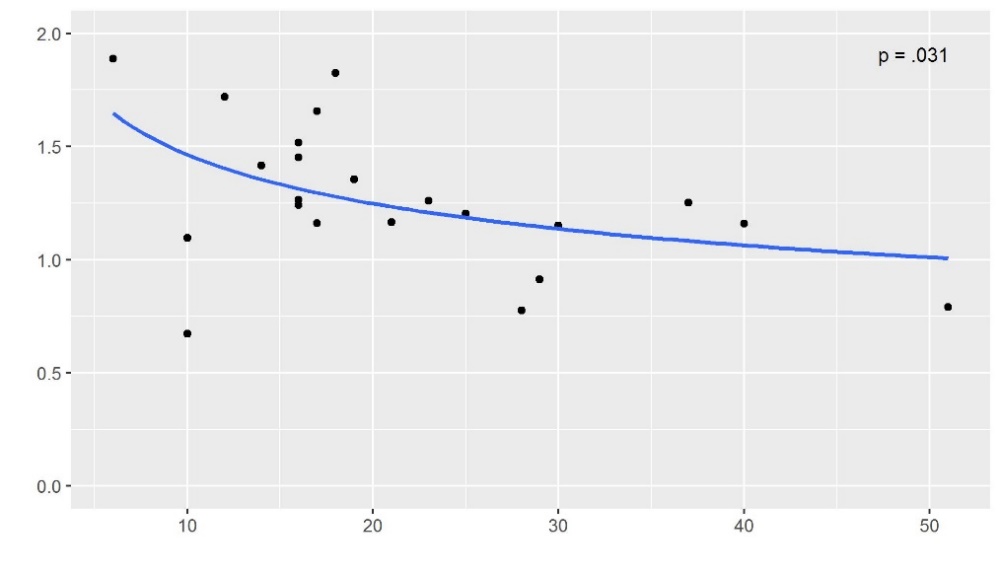 | Putamen  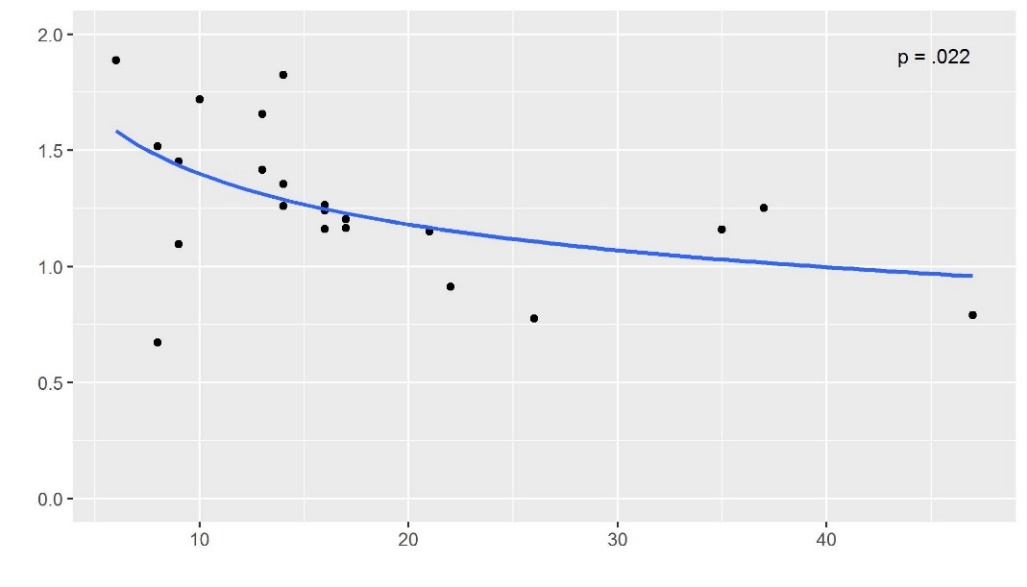 |  |
|  | SMS  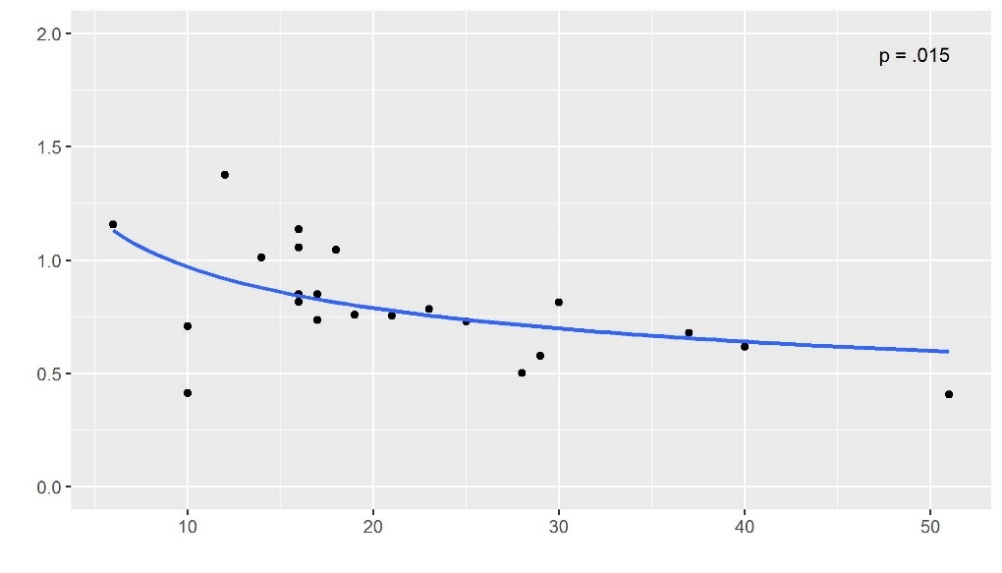 | SMS  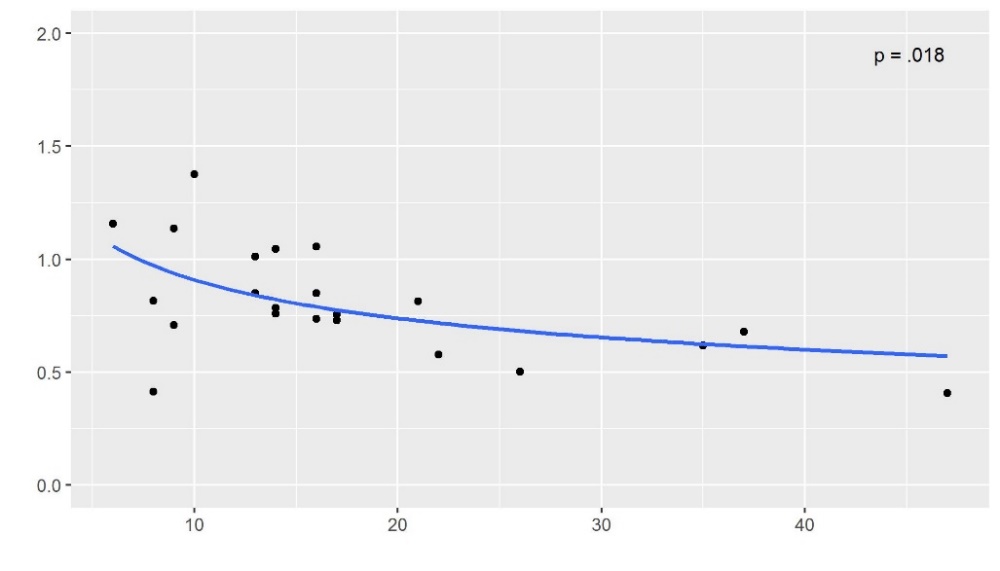 | |

|  | **MDS-UPDRS-III score incl. tremor** | **MDS-UPDRS-III score excl. tremor** |
| --- | --- | --- |

p-value cut-off for significance is .042, after correction for multiple comparisons with calculated meff for two regions (.05/1.178=.042)
